# Arthroscopic Treatment of Calcific Tendinitis of Gemellus Superior and Gemellus Inferior: A Case Report and Literature Review

**DOI:** 10.1111/os.13186

**Published:** 2022-02-13

**Authors:** Wen‐bo Yang, Qian‐kun Xu, Xing‐huang Liu, Prapti Bakhshi, Hong Wang, Zeng‐wu Shao, Chun‐qing Meng, Wei Huang

**Affiliations:** ^1^ Department of Orthopaedic Surgery, Union Hospital, Tongji Medical College Huazhong University of Science and Technology Wuhan China; ^2^ Department of Orthopaedic Surgery Central Hospital of Hefeng County Enshi China

**Keywords:** Arthroscopic treatment, Benign lesion, Calcific tendinitis, Gemellus, Hip pain

## Abstract

**Background:**

Tendon calcification is a common disease, and it could happen in the tendons of the shoulder, wrist, etc. However, tendon calcification in the superior and inferior gemellus is rare, and in this region is likely to be misdiagnosed.

**Case Presentation:**

Here, our case report first reported a 53‐year‐old female patient with an unusual case of calcific tendinitis of the gemellus superior and gemellus inferior muscles. The patient presented with severe pain in the right hip and lower extremities, not relieved using nonsteroidal anti‐inflammatory drugs (NSAIDs). The preoperative physical examination indicated an abnormality in the hip joint. Preoperative imaging confirmed the results of the physical examination and showed a right hip lesion. We did not make a definite diagnosis preoperatively but considered that the patient might have an osteochondroma. However, surgical findings indicated that the lesion was not in the hip capsule on subsequent arthroscopic surgery, which suggested that the preoperative diagnosis might be wrong. We opened the posterior capsule and found a “toothpaste‐like” lesion in the superior and inferior gemellus muscles' tendon. We thought this might be the calcified tendon. Then the arthroscopic surgery was finished to remove the lesion, and the removed tissue was sent to the pathology department for pathological examination. The pathological report confirmed the diagnosis of the calcified tendon. Postoperative follow‐up was conducted. The effect of the operation was noticeable. Postoperative symptoms were relieved.

**Conclusions:**

Calcification of the tendons of the superior and inferior gemellus muscles can be easily misdiagnosed, and the disease can be treated minimally with arthroscopy.

## Graphical abstract

We found a case of calcified tendonitis located in the superior and inferior Gemellus muscles that may be misdiagnosed as osteochondroma. An arthroscopy revealed that the focus was calcified tissue in the tendons that was "toothpaste‐like” instead of proliferative bony substances. We suggest that doctors should pay attention to distinguish benign bone tumor and soft tissue diseases such as tendon calcification in clinical treatment.

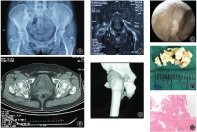

## Introduction

Calcification is a physiological process that occurs in bone tissue during bone growth. Calcification of tissues other than bone is pathological and is termed ectopic calcification. Ectopic calcification of a tendon is a typical manifestation of chronic tendinopathy. Calcific tendinitis occurs as a result of an accumulation of calcium hydroxyapatite in tendons^1^. At present, the etiology of calcific tendinitis is not clear, but genetic, traumatic, and local metabolic factors maybe related to calcium deposits and calcified tendonitis^2^, ^3^, ^4^. Calcific tendinitis is also associated with cortical bone erosion^5^, ^6^. Calcification of tendons usually occurs in the rotator cuff muscles of the shoulder joint. But it can also occur in other tendons throughout the body, such as the tendon of the hand, wrist, hip, and others^7^, ^8^, ^9^. Up until now, some cases of tendon calcification occurring in the hip joint have been reported, including the rectus femoris^10^ and adductors tendons^18^, etc. The use of technologies such as X‐ray and CT have allowed the diagnosis of tendon calcification to become much more accurate and have significantly reduced the rate of misdiagnosis. The precise diagnosis of tendon calcification is partly due to the tendons commonly involved in the pathology being relatively superficial, making it easier to recognize the calcified lesions on X‐ray or CT films. However, the diagnosis of tendon calcification within the hip joint is much more difficult as these tendons are relatively deeper and surrounded by many anatomical structures. Another issue in diagnosing calcific tendinitis of the hip joint is that it does not have any specific clinical manifestations that may help differentiate it from other pathologies within the hip joint. Hence, the misdiagnosis of calcific tendinitis of the hip joint is quite common and interferes with selecting an appropriate treatment plan. This case report describes the diagnosis, arthroscopic surgical treatment, and rehabilitation of a patient with calcific tendinitis of the hip joint.

## Case Report

### History

A 53‐year‐old female patient presented with pain of the right hip joint and lower limb for about a half year. The pain experienced a sudden onset when walking with weight‐bearing, with no apparent cause of inducement, and had been consistent over the past six months. There were no obvious sensory disturbances experienced in the affected joint or limb. In the month before the arthroscopic treatment, the severity of the pain had increased significantly and limited the patient's activities of daily living. Subjective hip pain was measured with a visual analog scale (VAS) pain score. The preoperative pain score was 6. After the diagnosis, the patient took non‐steroidal anti‐inflammatory drugs, which were not successful in relieving the symptoms. The patient then chose arthroscopic surgical treatment at the suggestion of the surgeon.

### Physical Examination

Active and passive movements in all directions of the right hip showed significant pain. The range of motion of the right hip was limited, especially when pronated and adducted, compared to the contralateral hip joint. The Patrick test and Lasegue's sign were positive on the right side and negative on the left. These tests further confirmed the presence of an abnormality in the right hip joint. There was no swelling of the affected hip joint. The musculoskeletal and neurological systems were normal on examination but positive of Lasegue's sign.

### Laboratory Examination

Blood routine showed a mildly elevated leukocyte count at 5.41 G/L, percentage of neutrophils at 39.19%, and percentage of lymphocyte at 50.9%. Rheumatoid factor test (RF), anti‐streptolysin O test (ASO), glucose, electrolytes, and liver and kidney functions were normal.

### Preoperative Imaging Examinations

**Fig. 1:**
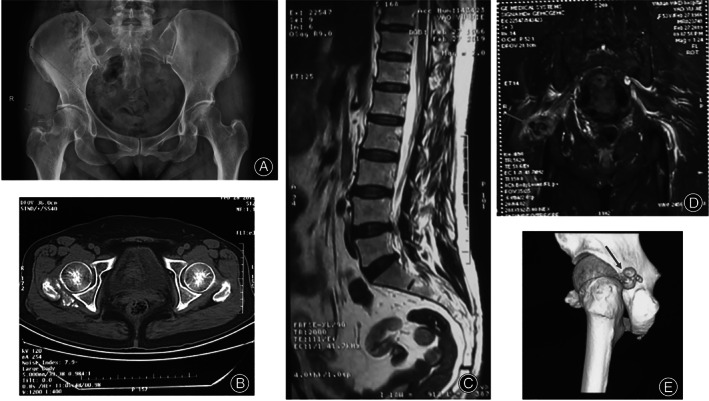
Preoperative imaging examination of the patient's lower back, pelvis and hip joint. (A) X‐ray examination of the pelvis in normal position; (B) CT of the right hip; (C) MRI of lumbar vertebrae; (D) MRI of hip; (E) three‐dimensional CT of the pelvis. In (A, B, and E) irregular cartilaginous bone and some nodular, posterior to the lateral side of the right hip joint, which adjacent to the greater trochanter of the right femur could be seen, as shown by the arrow. In (C) mild disc herniation in L_2‐3_, L_4‐5_, and L_5_‐S_1_ was observed, mainly L_5_‐S_1_ herniation accompanied by disc degeneration. In (D) abnormal signals in the superior and inferior gemellus regions of the right hip joint could be seen. Combined with CT results, synovial osteochondromatosis with peripheral soft tissue edema was considered.

High‐density shadow of the upper margin of the right femoral neck could be seen in the preoperative orthotopic X‐ray of the hip joint. MRI of the lumbar vertebrae showed mild herniation of the L_2‐3_, L_4‐5_, and L_5_‐S_1_ intervertebral discs, along with a degeneration of the L_5_‐S_1_ intervertebral disc. The hip MRI showed an abnormal signal in the superior and inferior gemellus regions of the right hip joint. Irregular perichondral bone in the external‐posterior part of the right hip joint along with nodular changes was found in the CT scan of the hip. The lesion was visible adjacent to the greater trochanter of the right femoral, which may indicate osteochondroma. The aforementioned images can be seen in Fig. 1.

### Treatments

**Fig. 2:**
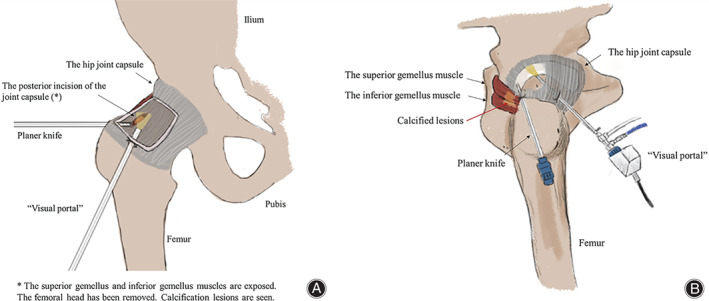
Schematic figure of surgical portal and lesion. (A) The frontal view of the surgical portal; (B) the lateral view of the surgical portal and lesion. The anterolateral portal of the hip joint was used as the “visual portal.” The planer knife was inserted through the posterolateral portal and the posterior capsule was planned to reveal the superior gemellus and inferior gemellus. The calcified lesion was then excised.

An arthroscopic surgical intervention was planned after the conservative treatment failing to relieve the patient's symptoms. According to the location of the lesion, the anterolateral and posterolateral portals of the hip joint were designed as surgical portals. The relationship between the anterolateral and posterolateral portals of the hip arthroscopy and the location of the lesion is shown in Fig. 2A,B. The anterolateral portal, as a “visual portal,” was mainly to observe the lesions. And the posterolateral portal was mainly to perform the corresponding surgical procedures. The greater trochanter of the femur was a reference for selecting location points for anterolateral and posterolateral portals. The anterolateral portal was approximately 2 cm in front of the greater trochanter of the femur and the posterolateral portal was approximately 2 cm above the posterior of the greater trochanter of the femur. The two anchoring points were about 4 cm apart. The patient was placed supine with the 30° abduction and 10° internal rotation of the hip joint. During the surgery, an anterolateral portal was first used to insert a 30° hip scope into the right hip cavity, which would provide a clear view of the hip cavity and the calcified gemellus superior and gemellus inferior tendons near the greater tuberosity. Unexpectedly, we did not find the osteochondroma lesions in the joints, which implied that the lesion might be outside the joint.

**Fig. 3:**
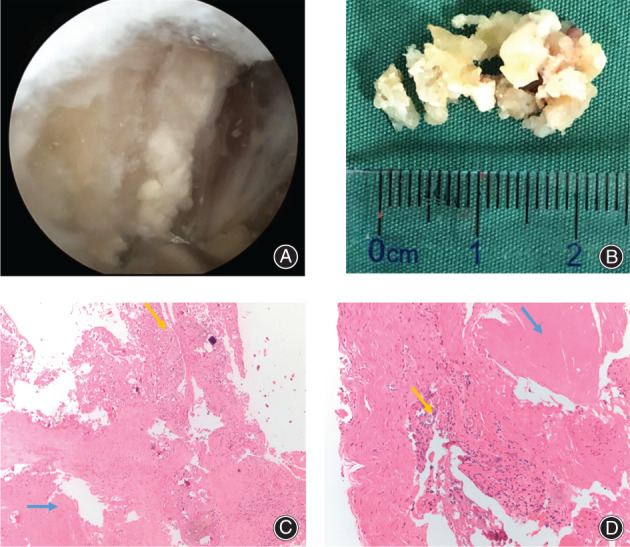
Direct view of lesion tissue and pathological findings. (A) The toothpaste‐like tissue seen intraoperatively; (B) the calcified tendon specimens; (C, D) pathology at low power and high power; the results mainly show hyperplasia of fibrous tissue with hyaline degeneration (blue arrow). There is also a small amount of synovial tissue hyperplasia with calcification in some areas (yellow arrow).

Second, we established the posterolateral portal with an epidural puncture needle for positioning. The posterior joint capsule was incised using radiofrequency ablation and a planer knife with a depth of approximately 8 cm. Then the gemellus superior and gemellus inferior were exposed, and the degenerated tendons were debrided medially, revealing a white, soft, toothpaste‐like matter (Fig. 3A).We found that the calcification occurred at the tendon body rather than the termination site through arthroscopic observation. Considering the pathological properties of tendon calcification, we only removed the lesion but did not remove the tendon, and the tendon‐bone structure was preserved. We collected the calcified tendon specimens from the hip cavity (Fig. 3B). We carried out the pathological examinations on the tendon specimens, and it confirmed that the calcified tendon specimens consisted of calcium hydroxyapatite crystals combined with proliferative fibrous tissue (Fig. 3C,D).

### Postoperative Physical Examination and Imaging Examination

**Fig. 4:**
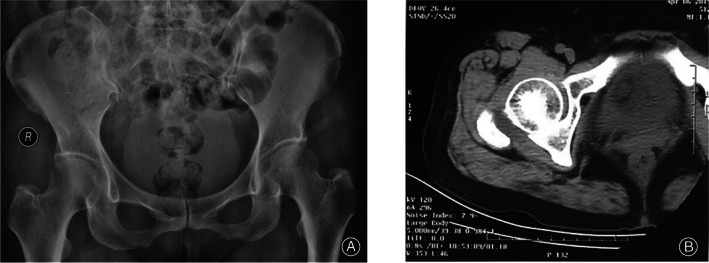
Imaging results. (A) Orthotopic X‐ray of the hip joint after surgery; (B) the postoperative CT of the hip joint, which suggested that the same lesion shown on X‐ray and CT before surgery was not found after surgery. The results indicate that the lesion has been removed by the surgical procedure.

Postoperatively, we could observe clear improvement sign in the patient. One week after surgery, a physical examination of the right hip showed a negative Patrick sign, as well as no limitation of activities of daily living. Moreover, the Lasegue sign was also negative. The VAS score dropped to 2. Three‐week post‐surgery, the VAS score dropped to 0. An orthotopic X‐ray examination of the hip joint was performed after surgery (Fig. 4A). The postoperative CT of the hip joint is shown in Fig. 4B. No high‐density shadows could be seen in the upper margin of the right femoral head.

### Prognosis of Patient

The patient recovered well after surgery without complaining of obvious discomfort. We followed the patient up for one year. During follow‐up, the patient recovered well without any complications. The patient no longer had significant pain, and the movement of the affected limb was normal. The physical examination of the hip joint showed no obvious abnormalities. The patients expressed reasonable satisfaction.

## Discussion

Calcific tendinitis is a painful condition that can affect many different joints in the body, such as the shoulder, wrist, and hip joints, and sometimes occurs in the neck, resulting in severe pain^11^, ^12^, ^13^, ^14^, ^15^. Patients in the clinic commonly have calcific tendinitis of the rotator cuff muscles in the shoulder joint^16^, ^17^. However, the calcification of tendons in other joints is rare. As of now, the etiology of this disease is not definitively known, although some evidences have suggested local metabolic factors, trauma, genetic factor, and others are possible causes of this disease^2^, ^3^, ^4^. As mentioned earlier, cases of tendon calcification of the hip joint are sporadic, and therefore, the use of arthroscopy to treat the disease is not widespread. So far, in our research, we have found case reports describing calcification of the rectus femoris tendon^10^ and the adductor tendons^18^ being affected in the hip joint by this disease. We reviewed the relevant articles on tendon calcification near the hip joint and listed the lesion sites and treatment methods mentioned in the relevant articles in Table 1. Even though the number of cases encountered is few, calcific tendinitis of the hip joint needs to be concerned as it can result in severe clinical manifestations, which may require the operation of the patients^19^, ^20^.

**TABLE 1:** A review of articles on tendon calcification near the hip joint**

First author	Site of lesion*	Main Treatment	Detailed method
Zini^10^	Rectus femoris	Arthroscopic excision	Using arthroscopy.
Lee^21^	Rectus femoris	Extracorporeal shock wave therapy	Pressure pulses focused. A single session of ESWT. The frequency: a total of six times, and at an interval of 3–4 days.
Jo^22^	Gluteus Medius	Ultrasonography‐guided barbotage	One‐needle technique. Local anesthesia. The lavage of the calcified lesions: 0.9% saline.
Peng^20^	The origin of the rectus femoris; relevant intra‐articular lesions.	Arthroscopic treatment	Positioning: C‐arm machines and Kirschner wires. Anterior, anterolateral and posterolateral portals.
Braun‐Moscovici^19^	The rectus femoris muscle	Local injections; anti‐inflammatory drugs	The following methods are mentioned: NSAID—Rofecoxib; Methylprednisolone in combination with lidocaine; Local corticosteroid injection.
Hong^23^	The attachment site of rectus tendon	Ultrasound‐guided injection	Identify the lesion using a transducer. Mepivacaine with triamcinolone acetate was injected. Gave it again a week later.
Almedghio^24^	Gluteus Medius	Analgesia and NSAIDs/steroids	The drugs were administered by injection.
Sakai^25^	Gluteus medius	NSAIDs	The patient was treated conservatively with NSAIDs.
McLoughlin^26^	The direct head of the rectus femoris	Ultrasound‐guided percutaneous irrigation of calcific tendinopathy (US‐PICT)	The lesions were located using US‐PICT. Local anesthesia (1% lidocaine). Intermittent pulse lavage of the lesion area using 3 mL of 1% lidocaine.
Williams^27^	The femoral insertion of the gluteus maximus muscle	Open operation	A standard lateral approach. Excising the lesion. Repairing and augmenting local anatomical structure.
Comba^28^	The rectus femoris	Endoscopic treatment	Anterolateral portal and proximal accessory portal. Remove the lesion using the burr.
Choudur^29^	The gluteus maximus	Local anesthetic and corticosteroid	Local anesthetics and corticosteroid were injected through a puncture guided by fluoroscopy or CT.
Huang^30^	The gluteus maximus	Ultrasound‐guided; methylprednisolone and bupivacaine	Methylprednisolone and bupivacaine were injected into the gluteus maximus tendon insertion *via* ultrasound‐guided injection.
Kandemir^31^	Gluteus medius and minimus	Endoscopic treatment	Two portals 2 cm apart. The lesion was identified by endoscopy and removed by motorized shaver. Bony overgrowth and inflammatory changes were resected.
Vereecke^32^	The gluteus medius	Ultrasound‐guided needle lavage and anesthetic /corticosteroid injection	Using ultrasound to locate the lesion and needle. The lavage of lesion: 1% Linisol. Infiltration: 40 mg depoMedrol dissolved in 4 mL 0.5% marcaine.
Lin^33^	The gluteus medius	Acupuncture and small needle scalpel therapy	Small needle knife and acupuncture at 1, 2 weeks. Continued acupuncture once a week for 12 sessions.
Yang^34^	The rectus femoris	Endoscopic treatment	Anterolateral and proximal ancillary portals. Positioning: C‐arm machine and spinal needle. Removed the lesion using motorized shaver. Debrided the degenerated tendons.

^*^ Muscles or other structures related to the lesion are indicated. ** Table contents were taken from the original articles and may be duplication.

Based on our research, this clinical report is the first to describe a case of calcific tendinitis of gemellus superior and gemellus inferior muscles. It has been suggested that calcification around the joints can cause severe pain^35^. As these symptoms are atypical, the disease may be easily misdiagnosed as osteochondroma, gout, ossifying myositis, bone hyperplasia, etc. As in this case, despite taking a detailed history of our patient and carrying out a proper physical examination, we still misdiagnosed the disease as osteochondroma. Even preoperative imaging findings supported the diagnosis of osteochondroma. The patient had a lumbar MR examination preoperatively, and it showed a slight lumbar disc herniation. However, in our clinical experience, such a mild lumbar disc herniation does not usually result in an overt positive straight‐leg elevation test. We fully communicated with the patient preoperatively that the cause of the lower extremity pain could be related to the herniated disc but could also be due to the lesion in the hip joint, or both as these lesions can irritate the sciatic nerve, which can cause the positive Lasegue sign. The patient understood and agreed with us. One week after surgical treatment, the patient showed negative on the Lasegue test, indicating that the leg symptoms were sciatic nerve stimulation due to calcification of the hip joint. If the lumbar disc herniation is the primary diagnosis of this patient, the therapeutic effect will be minimal. Therefore, a hip examination is also of great significance for the identification of lower limb pain. If the patient has disc herniation disease, it will also cause some difficulties in the final diagnosis of the primary disease and lead to deviation in treatment.

Many operative and non‐operative treatment strategies are available for calcific tendinitis, such as nonsteroidal anti‐inflammatory drugs (NSAIDs), physical modalities, drug injections, and arthroscopic surgery^12^. Conservative treatments for tendon calcification are ineffective in many cases, requiring the patients to undergo surgery. Arthroscopic surgery is a good option for surgical intervention as it is a minimally invasive method to remove the calcified lesion. Compared with conservative treatments, arthroscopic surgery can often enable patients to achieve rapid recovery.
